# Dynamic and history of methane seepage in the SW Barents Sea: new insights from Leirdjupet Fault Complex

**DOI:** 10.1038/s41598-021-83542-0

**Published:** 2021-02-23

**Authors:** Claudio Argentino, Kate Alyse Waghorn, Sunil Vadakkepuliyambatta, Stéphane Polteau, Stefan Bünz, Giuliana Panieri

**Affiliations:** 1 CAGE - Centre for Arctic Gas Hydrate, Environment and Climate, Department of Geosciences, UiT The Arctic University of Norway, 9037 Tromsø, Norway; 2 Oslo Innovation Center, VBPR - Volcanic Basin Petroleum Research, 0349 Oslo, Norway; 3 Institute for Energy Technology, 2007 Kjeller, Norway; 4 SurfExGeo, 0776 Oslo, Norway

**Keywords:** Carbon cycle, Climate sciences, Marine chemistry

## Abstract

Methane emissions from Arctic continental margins are increasing due to the negative effect of global warming on ice sheet and permafrost stability, but dynamics and timescales of seafloor seepage still remain poorly constrained. Here, we examine sediment cores collected from an active seepage area located between 295 and 353 m water depth in the SW Barents Sea, at Leirdjupet Fault Complex. The geochemical composition of hydrocarbon gas in the sediment indicates a mixture of microbial and thermogenic gas, the latter being sourced from underlying Mesozoic formations. Sediment and carbonate geochemistry reveal a long history of methane emissions that started during Late Weichselian deglaciation after 14.5 cal ka BP. Methane-derived authigenic carbonates precipitated due to local gas hydrate destabilization, in turn triggered by an increasing influx of warm Atlantic water and isostatic rebound linked to the retreat of the Barents Sea Ice Sheet. This study has implications for a better understanding of the dynamic and future evolution of methane seeps in modern analogue systems in Western Antarctica, where the retreat of marine-based ice sheet induced by global warming may cause the release of large amounts of methane from hydrocarbon reservoirs and gas hydrates.

## Introduction

Concerns have risen in the last decades regarding the potential effect of global warming on inducing and accelerating natural methane emissions from wetlands, permafrost and the ocean seafloor^1,2^. The Arctic has received particular attention as it is more susceptible to temperature changes compared to the rest of the globe^3^ and, in recent years, has recorded an alarming increase in mean annual surface air temperature^4,5^ associated with ice-cover reduction^6^. In this scenario, direct methane emissions into the atmosphere increase the total greenhouse gas budget^7–9^, inducing a positive warming feedback. Oceans appear to provide a minor contribution^10,11^, representing 3–6% of all natural emissions^12^, as most methane leaking from marine sediment is oxidized close to the seafloor^13^ and in the water column^14,15^ before reaching the atmosphere. The highest methane fluxes are reported from continental margins in near-shore coastal environments, where ebullitive gas transport limits the efficiency of the benthic filter and oxidation in shallow waters^9,16,17^. Shallow Arctic continental margins store a vast amount of carbon in the form of gas hydrate, a crystalline ice-like compound stable in specific temperature and hydrostatic pressure conditions^18^. Dissociation of gas hydrate through slow steady degassing is unlikely to significantly contribute to overall ocean methane outputs^1^, whereas catastrophic methane release related to blow-out events will most likely reach the atmosphere in shallow seas^19^. Future blow-out events may release into the water column up to 116–541 Gt of carbon stored in the Arctic^20,21^ due to global warming^1^, amplifying ocean acidification^7,22^. However, to date, the key processes and factors controlling the dynamics of methane emissions at the seafloor remain highly uncertain^9^.

The aims of this study are to characterize the evolution and constrain the main mechanisms driving active methane seepage recently discovered along the Leirdjupet Fault Complex in the SW Barents Sea. The seep area was detected based on gas flares in the water column, as well as bacterial mats and gas bubbling on the seafloor imagery. We examined a total of three gravity cores and five video-guided multicores and employed an integrated approach including geochemical investigation of sediment, foraminifera, pore water and hydrocarbon gas in the sediment. The results of this study are relevant for better understanding the dynamics between deglaciation and methane leakage in modern analogue systems in Western Antarctica, where the retreat of marine-based ice sheet induced by global warming may cause the release of large amounts of methane from hydrocarbon reservoirs and gas hydrates.

### Study area

The Barents Sea is a natural laboratory to study the development and evolution of methane seepage systems, in particular by focussing on the interplay between sub-seabed geology (fault structures, stratigraphy, lithology) and glacial history (isostatic adjustments, glacial loading) in regulating seafloor methane emissions^23–25^. Present-day seafloor morphology reflects the effect of glacial processes during multiple phases of ice sheet retreat and advance since the Pliocene^26,27^. The preserved structures on the seabed include mega-scale lineations and deep glacial valleys carved out by ice-streams during the last glacial advance and numerous iceberg ploughmarks and pits^26,28,29^. Seafloor features related to fluid expulsion processes e.g. pockmarks and craters are widespread and their formation, in some cases, is related to the destabilization of methane hydrates^19,30^ fed from leaking Mesozoic hydrocarbon reservoirs^31–34^. In addition, the Barents Sea is a paleo-analogue of the Antarctic^35^, and therefore may help better predict the evolution of the modern gas hydrate systems beneath the marine-based West Antarctic Ice Sheet.

**Figure 1:**
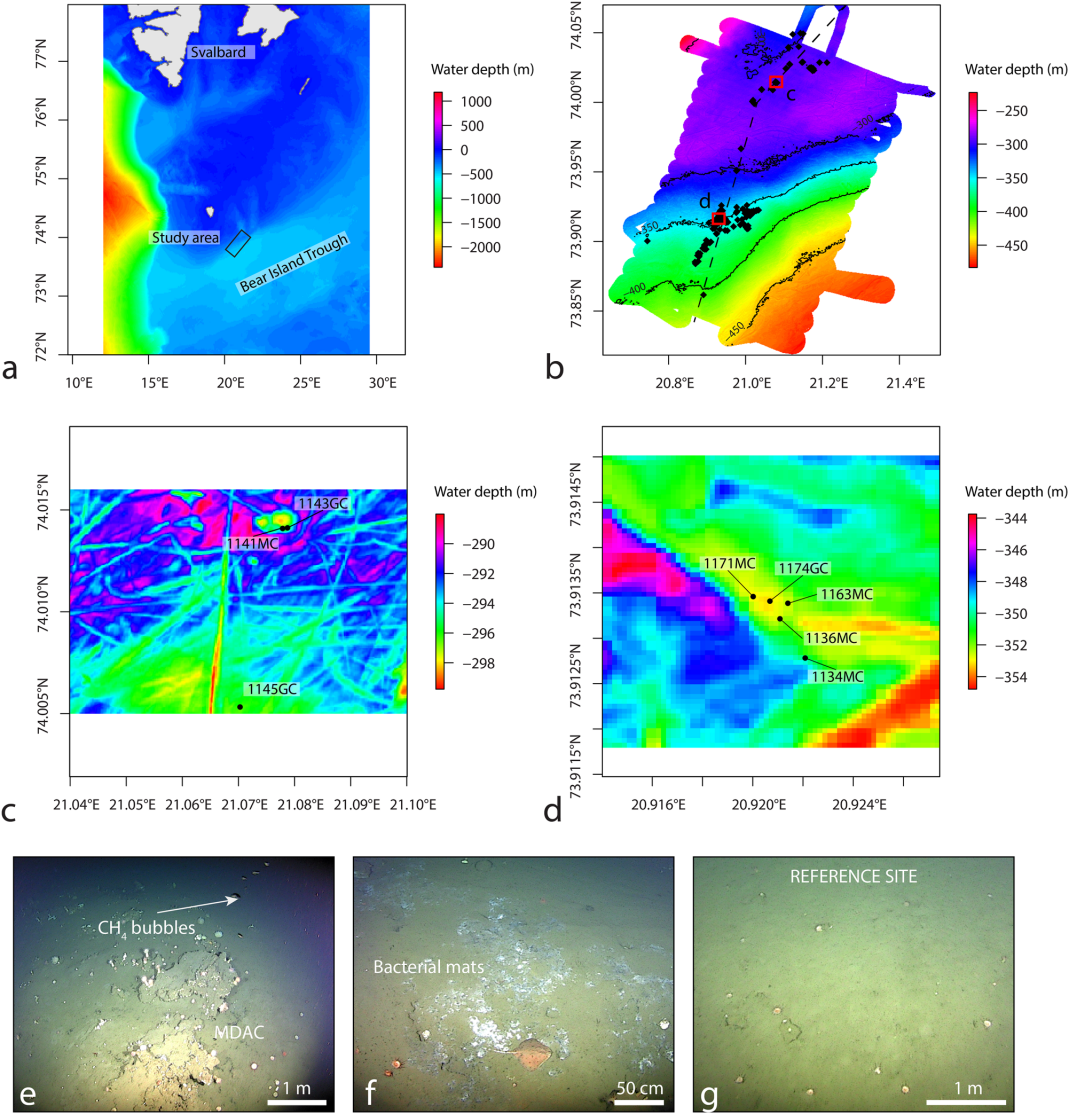
(**a**) Bathymetric map of the WesternBarents Sea showing the location of the Bear Island Trough and of the study area (box). (**b**) Distribution of gas flares (black polygons) around Leirdjupet Fault Complex (the dashed line marks the main fault). (**c**) Distribution of the sediment cores in the Pockmark area (cores 1141MC, 1143GC) and reference core 1145GC. (**d**) Distribution of sediment cores in the Ploughmark area. (**e**) Methane-derived authigenic carbonates (MDAC) and methane bubbles in the Pockmark area. (**f**) Bacterial mats in the Ploughmark area. (**g**) Non-seep area used as a reference site.

New seeps were discovered in two clusters along the Leirdjupet Fault Complex (LFC) on the northern flank of the Bear Island Trough (CAGE17-3 cruise report, http://cage.uit.no/cruise-logs), a transverse shelf trough carved by a Late Weichselian ice stream (Fig. 1a,b). These seeps were identified based on the presence of gas flares in the water column imaged as hydroacoustic anomalies that are rooted on the seafloor. Coring operations at water depth ranging from 295 to 353 m targeted the first (Pockmark area, Fig. 1c) and the second cluster (Ploughmark area, Fig. 1d), and a reference site located 400 m away from gas flares (Fig. 1c). The Pockmark area is characterized by the occurrence of methane-derived authigenic carbonates (Fig. 1e), whereas the Ploughmark area hosts several bacterial mats (Fig. 1f) and the reference site is barren of seep-related features (Fig. 1g).

## Results

### Chronology and lithofacies

**Figure 2:**
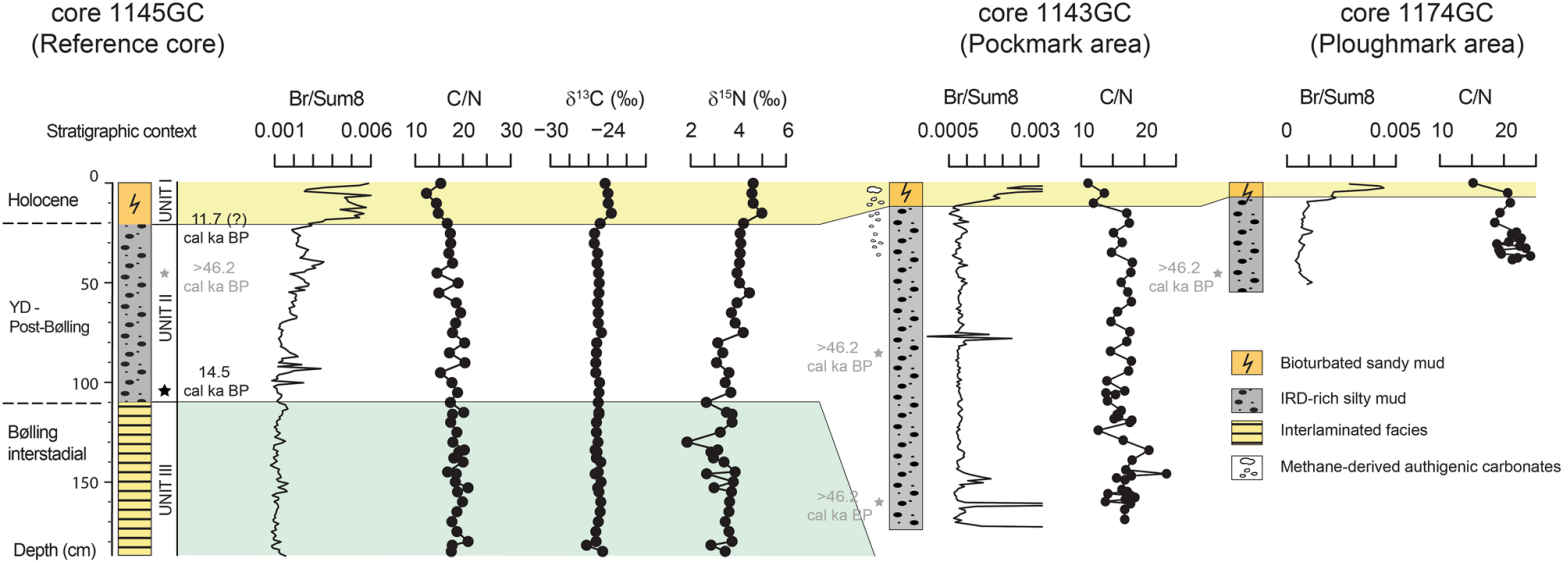
Chronostratigraphic model of reference core 1145GC and correlation with cores 1143GC and 1174GC. Core 1145GC has been subdivided into three lithostratigraphic units and interpreted based on a radiocarbon tie point at the base of Unit II and integrated with paleoproductivity proxies and organic matter parameters (Br/Sum8, C/N ratio, δ^13^C, δ^15^N) to identify the Holocene sediment interval (Unit I). The typical marine organic matter is composed of plankton and algae and is characterized by C/N values between 4 to 10, δ^13^C_org_ between −23 and −16‰ and δ^15^N as high as 7‰^78–80^. Terrestrial organic matter mainly includes material from vascular land plants showing C/N values above 20^79^, lighter δ^13^C values ranging from − 29.3‰ to − 25.5‰^81^ and δ^15^N close to atmospheric nitrogen composition (~ 0‰)^78^. Mollusc shells collected from Unit II in cores 1145GC, 1143GC and 1174GC provided radiocarbon ages > 46,220 cal years BP and cannot be used for correlation. Unit II and Unit III represent the Younger Dryas – post Bolling period and the Bolling interstadial, respectively. IRD = Ice-rafted debris.

The chronostratigraphic model was first established on the 185 cm-long reference core 1145GC (Fig. 2; Supplementary Fig. S1) based on lithofacies (grain size composition and sedimentary features), a radiocarbon tie point obtained from foraminiferal tests (Fig. 2), and correlations to sediment cores from adjacent areas^36–38^. The lithofacies Unit I, Unit II and Unit III (Fig. 2) identified in core 1145GC reflect the regional environmental evolution of the SW Barents Sea during Late Weichselian deglaciation marked by sediment deposition through meltwater plumes and iceberg rafting.

Unit I is preserved in the upper 20 cm of the core 1145GC and represents Holocene sedimentation (~ 11.7 cal ka BP)^39^ with bioturbated sandy mud, light brown in colour. The geochemical composition shows a clear change in inputs of organic matter compared the rest of the core. Enhanced marine productivity in this unit is indicated by high bromine concentration (Br/Sum8)^40^ associated with low average C/N = 14.7 ± 1.6 (SD; n = 5) and δ^13^C_org_ = −24.1‰ ± 0.4‰ (SD; n = 5), and high δ^15^N = 4.6‰ ± 0.3‰ (SD; n = 5) (Fig. 2).

Unit II in core 1145GC covers the sediment interval 20–110 cm and consists of dark grey silty mud with ice-rafted debris (IRD). The IRD cause the magnetic susceptibility to display a random/chaotic pattern preventing direct detailed correlations with other cores (Supplementary Fig. S2). Unit II displays average C/N = 17.7 ± 1.7 (SD; n = 18), δ^13^C_org_ = −25.0‰ ± 0.2‰ (SD; n = 18) and δ^15^N = 3.7‰ ± 0.5‰ (SD; n = 18) (Fig. 2) suggesting an increased contribution from land-derived organic matter. Foraminiferal tests at the base of this unit yielded a radiocarbon age of 14,484 ± 333 cal years BP, thus ascribing deposition of Unit II to the Younger Dryas—post Bølling period. This unit is similar to Unit A1 described by Lucchi et al.^36^ from cores collected in the Storfjorden and Kveithola trough mouth fans and Unit 2 of Kaparulina et al.^38^ from cores collected in the Bear Island Trough.

Unit III (from 110 cm to the bottom of core 1145GC at 185 cm) consists of laminated grey silty mud and fine-grained sand, barren in fossils and bioturbation. The average C/N = 18.8 ± 1.2 (SD; n = 21), δ^13^C_org_ = −25.1‰ ± 0.3‰ (SD; n = 21) and δ^15^N = 3.3‰ ± 0.5‰ (SD; n = 21) (Fig. 2) together with the laminated sedimentation pattern are consistent with suspension settling from meltwater plumes in a glaciomarine environment^36,37^. Pebbles are rare, suggesting the presence of multi-year shorefast sea ice hindering iceberg formation^41^. This unit was deposited during the Bølling interstadial (15 cal ka BP) and is tentatively correlated to Unit A2 of Lucchi et al.^36^, Unit 3 of Kaparulina et al.^38^ and Unit Ib of Pau et al.^37^.

Chronostratigraphy and full geochemical results for the other cores from the Pockmark and Ploughmark areas are reported in Supplementary Table S3.

### Sediment geochemistry: barium, calcium and sulfur

**Figure 3:**
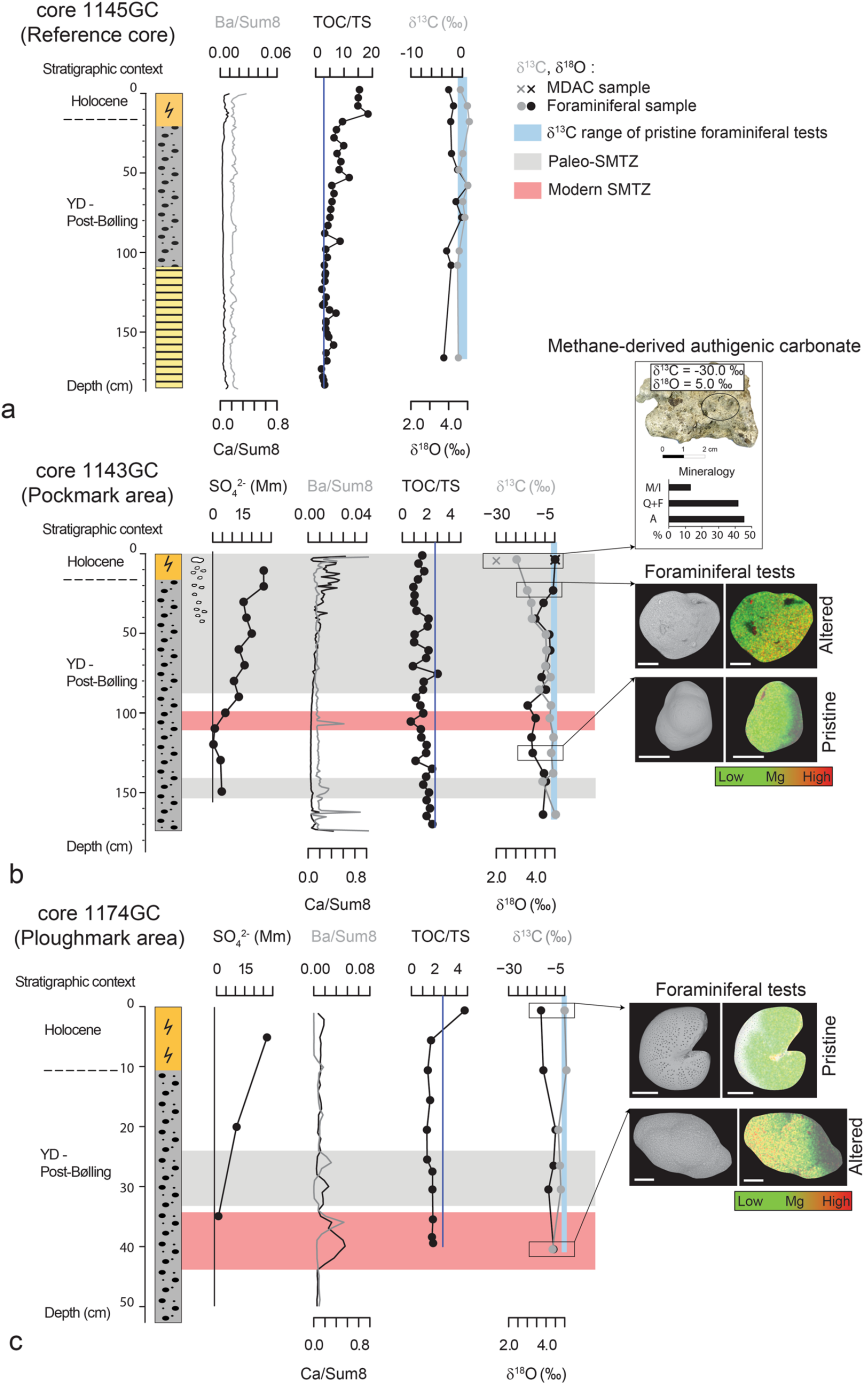
Pore water sulfate concentration and geochemical characterization of gravity cores 1145GC (**a**), 1143GC (**b**) and 1174GC (**c**). X-Ray Fluorescence profiles of barium (Ba), calcium (Ca), sulfur (S) along the three cores are compared with TOC/TS values and δ^13^C and δ^18^O composition of foraminiferal tests and one MDAC sample (core 1143GC) in order to constrain AOM-impacted sediment intervals. Barium peaks at 163 cm and 175 cm in 1143GC are analytical artifacts due to the presence of voids on core surface at the bottom of the core. SEM–EDS observations of foraminifera are also reported, from top to bottom: Cassidulina neoteretis, *Cassidulina reniforme*, *Melonis barleeanum* and undetermined foraminifera (high degree of alteration). Solid vertical line in the TOC/TS plots indicates the threshold value 2.8 used to distinguish background TOC/TS values (> 2.8)^42^ from AOM-impacted sediment intervals marked by an excess sulfur (TOC/TS < 2.8)^43,48^. Vertical shaded area (light blue color) in the δ^18^O plots indicate the usual range for unaltered foraminiferal tests^45,47^. The location of the modern SMTZ is highlighted with a horizontal red area (b, c), whereas paleo-SMTZs are reported in grey color. M/I = mica/illite; Q + F = quartz + feldspars; A = aragonite. (The reader is referred to the Web version of this article for interpretation of the references to color in this figure legend).

Semi-quantitative geochemical analysis via X-Ray Fluorescence (XRF) spectroscopy were conducted on reference core 1145GC and gravity cores 1143GC (Pockmark area) and 1174GC (Ploughmark area). The down-core profile of barium (expressed as Ba/Sum8) and calcium (expressed as Ca/Sum8) are shown in Fig. 3 and are used as proxies for barite and carbonate contents, respectively (the normalization procedure is described in the methods section). Reference core 1145GC shows relatively constant down-core barium and calcium contents (Fig. 3a). In core 1143GC, barium enrichments are found within the interval 0–4 cm, at 107 cm and at 147 cm (Fig. 3b). Calcium shows very high counts in the uppermost 40 cm of the core. In core 1174GC, high barium contents occur between 24 and 28 cm and between 33 and 39 cm, whereas calcium peaks are located between 28 and 34 cm and between 35 and 44 cm (Fig. 3c). In reference core 1145GC, Total Organic Carbon (TOC) and Total Sulfur (TS) follow a down-core decreasing trend and converge to the predicted TOC/TS diagenetic value of 2.8^42^ at 120 cm (Fig. 3a). In core 1143GC, TOC/TS values are consistently lower than 2.8, indicating excess sulfur in the sediment (Fig. 3b). In core 1174GC, TOC/TS values lower than 2.8 are found throughout the sediment core except for a value of 4.8 measured in the uppermost sample near the seafloor (Fig. 3c).

### Carbonate geochemistry and mineralogy: foraminifera and authigenic concretions

In core 1145GC, δ^13^C values of foraminiferal samples range between −0.9 and 1.3‰, whereas δ^18^O values range between 3.7 and 5.0‰ (Fig. 3a). Core 1143GC (Pockmark area) shows δ^13^C values between −19.5‰ and 0.3‰, and δ^18^O values between 3.6‰ and 5.0‰ (Fig. 3b). Most depleted δ^13^C values (< −10‰) are measured in the uppermost 40 cm of sediment in correspondence of macroscopic carbonate concretions. Foraminiferal samples collected from core 1174GC (Ploughmark area) yielded δ^13^C values between −6.6 and 1.0‰ and δ^18^O values between 3.7‰ and 4.5‰. Most depleted δ^13^C values in core 1174GC are found in sediment interval 20–30 cm (−3.4‰ < δ^13^C < −2.0‰) and at 40 cm (δ^13^C = −6.6‰) (Fig. 3c).

Carbonate concretions ranging in size from few mm to more than 5 cm were observed in cores 1143GC and 1141MC within the respective sediment intervals 4–30 cm and 16–28 cm (Fig. 2; Supplementary Fig. S1). A carbonate sample collected at 4–6 cm in core 1143GC yielded δ^13^C = −30.0‰ and δ^18^O = 5.0‰ (Fig. 3b). In core 1141MC, one sample collected at 18–20 cm shows δ^13^C = −31.0‰ and δ^18^O = 5.3‰ and one sample collected at 26–28 cm yielded δ^13^C = −31.5‰ and δ^18^O = 5.0‰ (Fig. 3; Supplementary Table S4). The mineralogical composition of carbonate concretions includes aragonite (core 1143GC) (Fig. 3b) and high-Mg calcite (core 1141MC) associated with a variable siliciclastic component consisting of quartz, feldspar, chlorite and mica/illite and minor amounts of gypsum(Supplementary Table S4).

### Pore water sulfate concentration and hydrocarbon gas composition

Sulfate concentration in pore water samples collected from all the examined cores define variable down-core trends (Supplementary Fig. S1). In cores 1136MC, 1143GC and 1174GC, sulfate is consumed to values close to 0 mM at 34 cm, 110 cm and 35 cm, respectively. In the other cores, sulfate concentration passes from a seawater value of around 28 mM close to the seafloor, to lower values of around 15 mM at the bottom of the cores.

**Figure 4:**
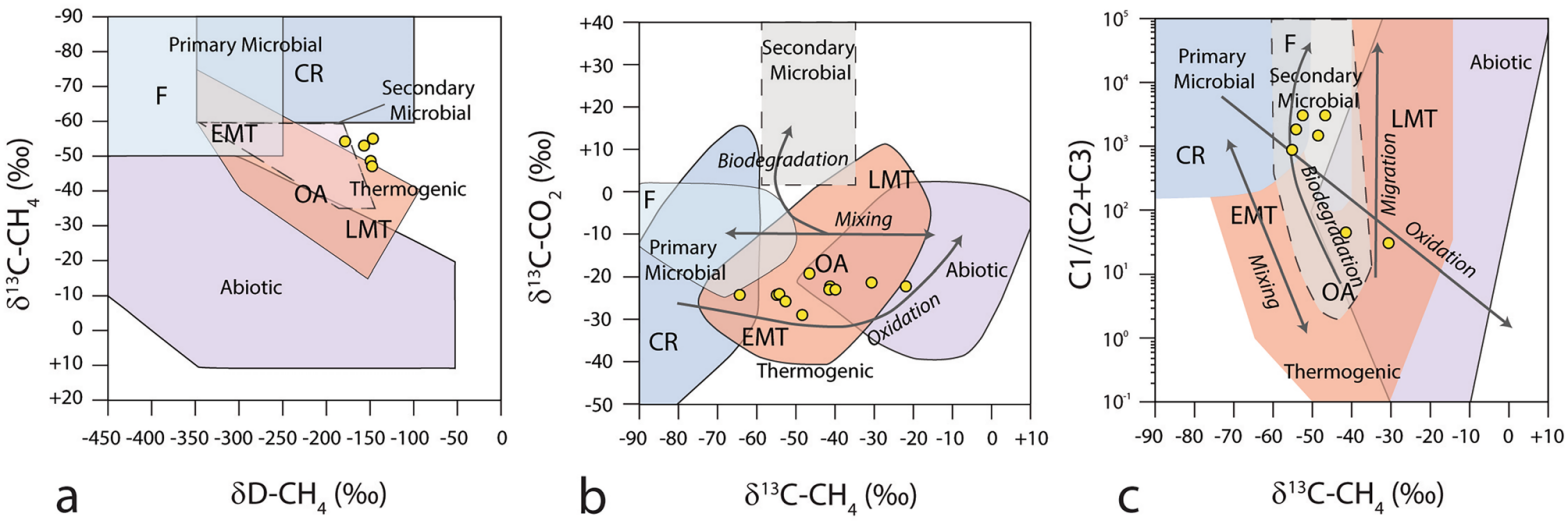
(**a**) Stable carbon (δ^13^C) and hydrogen (δ^2^H) isotope composition of methane from headspace gas analysis. Samples are reported in yellow dots. Genetic fields (CR -CO_2_ reduction, F—methyl-type fermentation, EMT—early mature thermogenic gas, OA—oil-associated thermogenic gas, LMT—late mature thermogenic gas) after Milkov and Etiope^59^. (**b**) Genetic diagrams of δ^13^C-C1 versus δ^13^C-CO_2_. Grey arrows indicate the main processes that affect the carbon isotopic composition of methane and CO_2_ in natural gases^59^. (**c**) Plot of δ^13^C-C1 versus the composition of light hydrocarbon components (C1/(C2 + C3) ratio). Grey arrows indicate the main processes affecting δ^13^C-C1 and the molecular composition of gases^59^.

The composition of gas samples collected from the bottom of sediment cores in the Ploughmark and Pockmark seepage areas shows variable methane concentrations. For total hydrocarbon gases + CO_2_ (THCG) concentrations > 10,000 ppm (n = 5), the methane fraction ranges from 96.9% to 99.3%, ethane from 0.03% to 0.12%, C6 + from 0.02% to 0.17% and CO_2_ from 0.61% to 3.07% (Supplementary Table S5). A total of 10 samples have low THCG contents (< 10,000 ppm) mainly dominated by CO_2_ (from 59.1% to 98.1%). Methane δ^13^C and δ^2^H composition ranges between −64.4 and −22.1‰, and between −179 and −148‰ (Fig. 4a,b), respectively. CO_2_ shows δ^13^C values between −29.9 and −19.9‰ (Fig. 4b). Gas wetness expressed as C1/ (C2 + C3) ranges from 25 to 2850 (Fig. 4c).

## Discussion

### A long history of methane emissions at Leirdjupet Fault Complex

The measurement of sulfate concentration in pore water samples collected along sediment cores enabled us to trace the current position of the Sulfate-Methane Transition Zone (SMTZ), corresponding to the depth within the sediment where sulfate is consumed below detection limit through Anaerobic Oxidation of Methane (AOM)^13^. In areas characterized by high methane flux, seawater sulfate diffusing into the sediment is readily consumed in the first few to tens of cm, resulting in a shallow SMTZ located close to the seafloor^13^. The AOM process is generally associated with the precipitation of methane-derived authigenic carbonates due to increased pore water alkalinity, and with solid iron sulfides. Therefore, we can track the position of modern and paleo-SMTZ by detecting sediment enrichments in these solid phases. During the diagenesis of marine sediments, total organic carbon and buried total sulfur co-vary linearly, converging to a TOC/TS ratio of ~ 2.8^42^, and values lower than 2.8 mark enhanced pyrite formation at the SMTZ^43^. The δ^13^C composition of carbonate tests of benthic and planktonic foraminifera collected from sediment samples represents an additional reliable proxy for AOM^44–46^. In fact, methane-derived carbonate precipitation on foraminiferal tests alter their bulk δ^13^C composition toward more depleted values, outside the usual range of seawater dissolved inorganic carbon (DIC) ranging from ~ −1 to ~ 1‰^47^. AOM-related geochemical anomalies generated at the SMTZ can be preserved in the sedimentary record, allowing the reconstruction of past episodes of methane oxidation associated with methane seepage^45,48^. The diagenetic history of the sediments may also alter the original isotopic signature of foraminiferal tests through dissolution/recrystallization of the original carbonate material. Diagenetic alteration of foraminiferal tests during burial may result in lighter δ^13^C values due to the incorporation of ^13^C-depleted carbon released into pore water by organic matter degradation processes^49^. However, the same specimens would also record an anomalously low δ^18^O composition due to temperature-dependent isotopic fractionation^49^. Therefore, any alterations to the original isotopic signature related to burial diagenesis can be easily ruled out during a preliminary diagenetic screening.

The geochemical composition of reference core 1145GC does not show any evidence for AOM (Fig. 3a). The geochemical composition of reference core 1145GC does not show any evidence of AOM and we argue that the modern SMTZ is located beneath 185 cm. Based on chronostratigraphic constraints we conclude that this sediment interval did not experience past episodes of methane seepage over the last 14.5 cal ka BP.

In core 1143GC from the Pockmark area, the pore water sulfate concentration profile highlights the presence of the modern SMTZ at 110 cm where its value reaches zero (Fig. 3b). At 107 cm, an isolated positive peak in barium is interpreted to mark the position of a diagenetic barite front formed by the interaction of upward migrating Ba-rich fluids with dissolved sulfate from seawater^50^. In this sediment layer, the carbon isotopic composition of foraminifera shows depleted δ^13^C values consistent with MDAC precipitation on their tests (Fig. 3b)^44–46^.

We also identified a paleo-SMTZs in core 1143G (Fig. 3b), where macroscopic authigenic carbonate crusts occur in the in the upper 40 cm, and remarkably depleted δ^13^C values in foraminifera (as negative as −19.5‰) are found down to 90 cm below the seafloor (Fig. 3b). In the stratigraphic interval 0–40 cm, the bulk organic matter δ^13^C composition shows an evident ^13^C depletion compared to the rest of the core (Fig. 3b). Bulk organic matter in sediments collected from the SMTZ may incorporate some ^13^C-depleted AOM-related biomass^51^. As we exclude the contribution from ^13^C-depleted terrestrial organic matter based on C/N and δ^15^N data (Supplementary Table S3), we propose that this sedimentary unit incorporated isotopically-depleted organic carbon of anaerobic methanotrophs^51–53^.

Carbonate concretions are interpreted as methane-derived authigenic carbonates based on their ^13^C-depleted carbon isotopic composition. MDAC-rich interval found in core 1143GC can be correlated to the interval 16–28 cm in core 1141MC located ~ 27 m away (Fig. 1c). However, MDACs from core 1141MC are dominated by high-Mg calcite whereas in core 1143GC they are composed of aragonite (Supplementary Table S4). Close to the seafloor, aragonite precipitation is favoured against calcite due to the inhibiting effect of SO_4_^2-^ and Mg^2+^ on calcite growth^54^, so we argue that the SMTZ in core 1143GC was shallower than in core 1141MC during time of carbonate formation. This interpretation is consistent with a small-scale spatial variability in methane flux as commonly observed at modern seeps^55^. Evident barite fronts appear in the uppermost 4 cm, at 107 cm (modern SMTZ) and at 143 cm. Barite formed at the upper limit of the SMTZ tends to re-dissolve when the SMTZ moves upward in the sediment column due to unsaturation^56^. Dissolution of barite fronts located deeper in the sediment column is retarded compared to barite enrichments located directly below the SMTZ^56^. We propose that the paleo-SMTZ at 143 cm recorded the oldest seepage event, followed by an increase in methane flux coupled with the rapid shoaling of the SMTZ close to the seafloor. During this transient phase, barite was rapidly buried and escaped significant dissolution while negligible MDAC precipitated on foraminiferal tests leaving a normal isotopic signature (Fig. 3b). That phase of intense methane flux was followed by a decreasing trend toward present-day conditions. This interpretation is supported by the presence of gypsum in a MDAC sample collected in 1141MC (Supplementary Table S4) that indicates a downward migration of the SMTZ toward present-day position^57^. Based on chronostratigraphic constraints we ascribe the seepage activity at this site to the Younger Dryas—post Bølling (< 14.5 cal ka BP) to Early Holocene.

In the Ploughmark area, the modern SMTZ is located close to the seafloor, at 35–44 cm (Fig. 3c; Supplementary Fig. S1). At this depth, the δ^13^C composition of foraminiferal carbonate is remarkably depleted (δ^13^C = −6.6‰) and tests show secondary Mg-rich carbonate overgrowth induced by AOM. In the same interval, barium and calcium show positive peaks reflecting sediment enrichments in barite and MDAC. Another barium-calcium couple is observed 10 cm above the modern SMTZ and is associated with a less depleted foraminiferal δ^13^C value (−3.4‰).

In summary, we interpret the interval 24–34 cm as a paleo-SMTZ. Other multicores from the Ploughmark area display sulfur enrichments in upper part of Unit II, in some cases also extending through Unit I (Supplementary Fig. S1), pointing to a similar history of methane seepage. Similarly to what is observed in the Pockmark area, the Ploughmark area recorded an overall decrease in seepage intensity through time which led to the deepening of the SMTZ. Based on chronostratigraphic constraints we can infer that methane fluxes started to decrease during the Holocene. Methane seepage is still active in both sites indicating a long history of methane emissions spanning several thousands of years and spread over much of the LFC area (Fig. 1b).

### Leakage of thermogenic-microbial gas from deep hydrocarbon reservoirs

The isotopic composition of methane (δ^13^C, δ^2^H) in headspace gas samples from sediment cores at LFC points to a predominant thermogenic origin of gas (Fig. 4a). The range of δ^13^C values of carbon dioxide (Fig. 4b) is compatible with both a microbial and a thermogenic source^58,59^ and allows us to rule out secondary petroleum biodegradation (Fig. 4B). Gas wetness (Fig. 4c) revealed the presence of a primary microbial gas component shifting the bulk methane δ^13^C composition toward more depleted values and increasing the C1/(C2 + C3) ratios. The isotopic composition of methane has been partially altered by microbial methane oxidation which consumes isotopically light methane and leads to a progressively heavier isotopic signature in the remaining C1 pool^59^ (Fig. 4b).

The SW Barents Sea petroleum system includes multiple source rocks located at stratigraphic intervals ranging from the Carboniferous to the Cretaceous^60^. The Hekkingen Formation (Upper Jurassic) is among the most prolific source rocks and consists of dark shales widespread over much of the SW Barents Sea. In the study area, the Hekkingen Formation potentially reached the oil window prior to Tertiary uplift and erosion^60^, while deeper Triassic and Permian/Carboniferous successions are gas mature or overmature^60^. Available information from hydrocarbon exploration wells (7321/7–1, 7321/8–1, 7321/9–1; e.g. http://factpage.npd.no) located 50–70 km south from the Ploughmark area reports a mature to highly mature kerogen in the Kapp Toscana Group and Adventdalen Group (Middle Triassic—Lower Cretaceous) and weak hydrocarbons shows in Triassic, Jurassic and Cretaceous intervals. The thermogenic gas component identified in the gas samples collected at LFC is compatible with a source rock located within the Mesozoic formations. During the ascent of hydrocarbon-rich fluids through fault structures, thermogenic gas mixed with shallower microbial gas possibly generated within Tertiary successions. The relatively heavy carbon isotopic composition of MDAC from sediment cores 1143GC (δ^13^C = −30.0‰) and 1141MC (δ^13^C from −31.5 to −31.0‰) is consistent with anaerobic oxidation of thermogenic and microbial methane. In fact, MDAC forming from microbial gas generally display δ^13^C values lighter than −45‰^23,25^. The results from carbonate geochemistry indicate that the composition of gas escaping from the present-day seafloor remained constant since the last < 14.5 cal ka BP and the same carbon reservoirs are still leaking today.

### Evidence for past gas hydrate destabilization

The oxygen isotopic composition (δ^18^O) of MDAC collected from the Pockmark area has been used to determine if gas hydrates contributed to past methane seepage in the SW Barents Sea. The δ^18^O composition of marine authigenic carbonate is primarily controlled by the composition of interstitial fluids in which it forms and by kinetic fractionation effects related to temperature and mineralogy^61^. At methane seeps, exotic fluids carrying a heavy δ^18^O signature may be derived from gas hydrate dissociation^62^ and clay mineral dehydration occurring at greater depth within the sediments^63^. We cannot rule out any contribution from the latter process as it is not possible to discriminate hydrate destabilization from clay dehydration by using δ^18^O of MDAC alone. However, the ascent of warm deep-sourced fluids would also have destabilized shallow gas hydrate reservoirs^64^, if present, as observed in other seepage areas worldwide^65–67^. Therefore, we first assessed whether MDAC formed in isotopic equilibrium with coeval seawater or from ^18^O-enriched fluids, then we calculated the stability of paleo-gas hydrates in the study area.

MDAC precipitated in equilibrium with bottom waters during the Late Weichselian deglaciation at ~ 15 ka would have recorded a δ^18^O of 4.7‰ (aragonite) or 5.6‰ (high-Mg calcite)^23^. MDAC formed later than ~ 15 ka, would carry a progressively lighter δ^18^O composition following temperature-dependent fractionation. In the present study, the aragonite sample collected from core 1143GC yielded a δ^18^O = 5.0‰, indicating a contribution from exotic fluids carrying a heavier oxygen isotopic signature compared to coeval seawater. Calcite samples in core 1141MC show values of 5.3‰ and 5.0‰, indicating a limited contribution from ^18^O-enriched fluids which resulted in an isotopic composition similar to the aragonite sample of core 1143GC. We calculated the gas hydrate stability zone (GHSZ) in the Pockmark area after 14.5 cal ka BP to validate whether gas hydrate dissociation may explain the analytical data (full parameters are reported in the methods section). Results indicated that pure methane hydrate was not stable in the Pockmark area. However, as frequently observed in present-day settings in the SW Barents Sea^68^, inputs of thermogenic gas to the GHSZ increase the stability field due to larger concentration of higher-order hydrocarbons. By assuming a molecular gas composition similar to the one measured in the Late Triassic Sandstones in well 7321 8–1 (http://factpage.npd.no) we obtained a 10–80 m-thick GHSZ, which indicates that the Pockmark area was located at the edge of gas hydrate stability. Therefore, we argue that MDAC from the Pockmark area recorded the influence of past gas hydrate destabilization. The majority of gas flares mapped in the water column at LFC are in the Ploughmark area (Fig. 1b) where pore water sulfate profiles show the shallowest SMTZ (Fig. 3c; Supplementary Fig. S1). The present-day edge of hydrate stability zone calculated using present-day average bottom water temperature of 2.5 ℃ crosses this area (10–60 m-thick GHSZ), however, to date there is no evidence for the presence of gas hydrates in the sub-seafloor to validate the results of this model.

**Figure 5:**
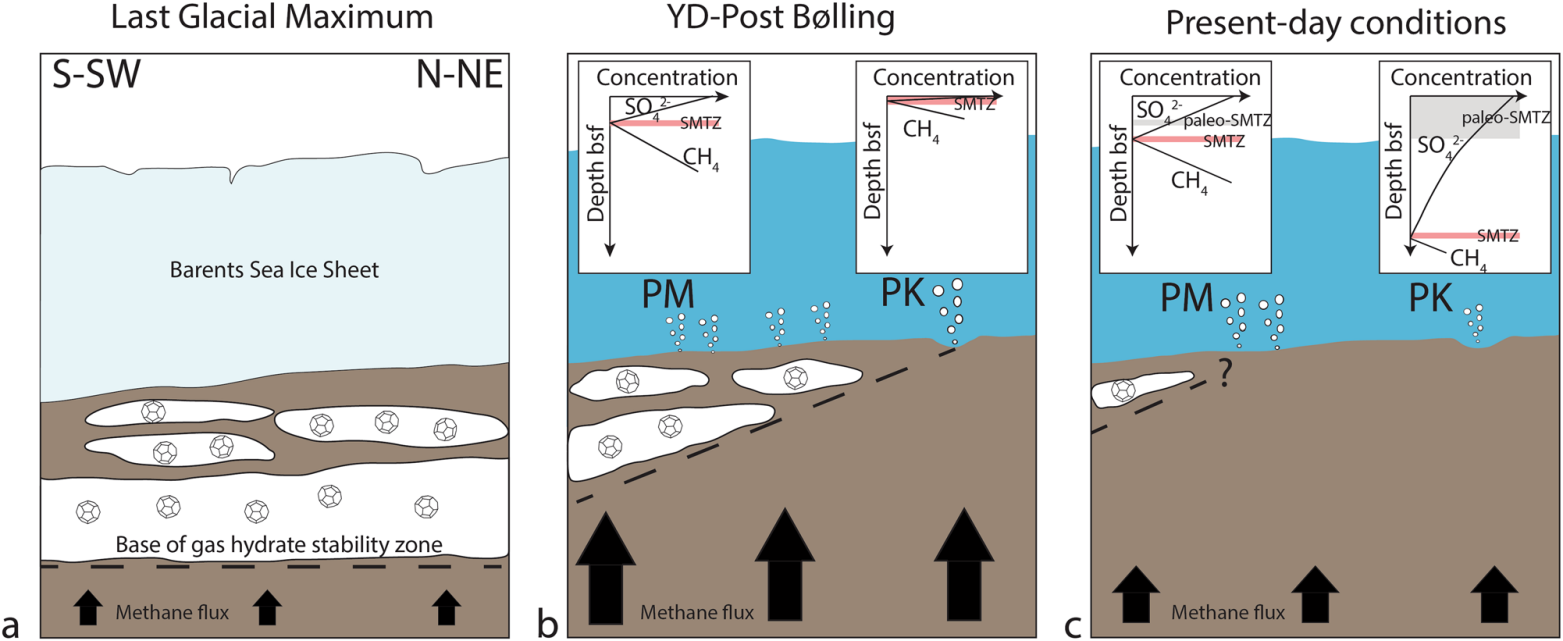
Schematic evolution of methane seepage at LFC after the LGM. (**a**) During the LGM, grounded ice sheet maintained gas hydrates stable for hundreds of meters below the seafloor. (**b**) The retreat of the ice sheet triggered hydrate destabilization and enhanced methane fluxes toward the seafloor causing the shoaling of the SMTZ. (**c**) An overall decrease in methane flux during the Holocene was recorded at both studied areas. PM = Ploughmark area; PK = Pockmark area. The modern SMTZs and paleo-SMTZs are highlighted in red and grey color, respectively. Not in scale.

Chronostratigraphic constraints indicate that hydrate destabilization started during the Younger Dryas—post Bølling period (later than 14.5 cal ka BP) and continued during part of the Holocene. Even though precise timing cannot be established through U/Th analysis due to the very high detrital content in the carbonates, our results are the first to validate the model proposed by Crémière et al.^23^ where widespread destabilization of gas hydrates in the SW Barents Sea^23^ is linked to the collapse of the Late Weichselian Ice Sheet. During the Last Glacial Maximum, the gas hydrate stability zone extended hundreds of meters below the seafloor due to the high hydrostatic pressure related to glacial loading^23^ (Fig. 5a). During the retreat of the Barents Sea Ice Sheet, the combination of isostatic uplift and influx of warmer Atlantic water induced hydrate destabilization at LFC and resulted in enhanced methane fluxes toward the seafloor. Shallow SMTZs developed both in the Ploughmark and Pockmark area. The latter was characterized by precipitation of MDAC close to the seafloor due to the proximity to the edge of gas hydrate stability (Fig. 5b). The data reported in the present study support the hypothesis of protracted hydrate-induced seepage on the order of thousands of years^23^. During the Holocene, seepage intensity progressively decreased due to the depletion of gas hydrate reservoirs and/or fault relaxation, causing the deepening of the SMTZs toward present-day positions (Fig. 5c).

Previous modelling estimates showed that at least 80% of methane bubbles rising from the seafloor at > 200 m depth dissolves in the water column before reaching the surface^15^ and up to 90% of methane released by gas hydrates is consumed within the sediment by microbial activity^69^. The estimated depth of the paleo seeps in the study area was close to present-day values^70^. Therefore, we can safely assume the fraction of methane released by hydrate destabilization and potentially reaching the sea-surface to be negligible when compared to the flux from other non-marine natural sources in terms of contribution to the atmospheric methane budget and influence on climate^1,71^. On the other hand, enhanced methane oxidation in the water column may have locally affected bottom water oxygenation and increased seawater acidification^7^. This process may potentially occur in the Arctic and Antarctic ice shelves that are disintegrating under current warming and amplify anthropogenic ocean acidification^7,22^, with dramatic implications on marine ecosystems.

Ocean warming recorded in the last decades over the western Antarctic shelves and associated with influx of relatively warm Circumpolar Deep Water close to the grounding zone is affecting the stability of the Western Antarctic Ice Sheet and may lead to an increase in methane emissions from the seafloor in our lifetime. As the Western Antarctic Ice Sheet and the Barents Sea Ice Sheet share similar geological and structural settings, the post-glacial history of methane emissions in the SW Barents Sea provides insights into the future evolution of seepage systems and gas hydrate reservoirs in Western Antarctica.

## Conclusions

Sediment cores collected from active seeps in the SW Barents Sea along the Leirdjupet Fault Complex represent a unique record of protracted methane emissions that started during the deglaciation of the Barents Sea Ice Sheet later than 14.5 cal ka BP. Geochemical gas composition from active seeps indicated a mix of thermogenic and microbial gases sourced from Mesozoic successions and overlying Tertiary deposits. Methane-derived authigenic carbonates with heavy δ^18^O signature recorded enhanced past seafloor methane fluxes linked to gas hydrate destabilization. Moreover, geochemical anomalies in sediment samples and foraminiferal tests highlighted an overall decrease in seepage intensity over the Holocene toward present-day conditions. Methane seeps at Leirdjupet Fault Complex provided a negligible contribution to atmospheric greenhouse gases with most of methane being oxidized within the sediment and in the water column. These results provide new insights into the dynamics and timescales of methane emissions in Arctic continental margins during deglaciation. In addition, this study highlights the urgent need to characterize the gas hydrate systems and monitor the seepage activity in the Western Antarctica, where the retreat of marine-based ice sheet induced by global warming may cause the release of vast amounts of methane from hydrocarbon reservoirs and gas hydrates.

## Methods

Sediment cores discussed in this study were collected during the cruise CAGE 18–4 on the R/V Helmer Hanssen to Leirdjupet Fault Complex, south-west Barents Sea. In this work we focused on three gravity cores CAGE 18–4 HH1143GC, CAGE 18–4 HH1145GC, CAGE 18–4 HH1174GC (referred in the text to as 1143GC, 1145GC and 1174GC) and five multicores CAGE 18–4 HH1134MC, CAGE 18–4 HH1136MC, CAGE 18–4 HH1141MC, CAGE 18–4 HH1163MC, CAGE 18–4 HH1171MC (referred to as 1134MC, 1136MC, 1141MC, 1163MC, 1171MC). A TowCam system mounted on the frame of the multicorer allowed the acquisition of real time video and guide the sampling. All following analyses have been performed at CAGE – Centre for Arctic Gas Hydrate, Environment and Climate located at The Arctic University of Norway (UiT), in Tromsø (Norway), except for AMS radiocarbon dating, pore water sulfate concentration analysis, gas analysis and mineralogical analyses.

### X-Ray Fluorescence analysis

XRF logging of cores 1143GC, 1145GC and 1174GC was conducted using an Avaatech XRF Core Scanner at 1 cm steps. Data were collected in multiple runs on each core applying different currents, voltages and filters: 10 kV, 1 mA, no filter; 30 kV, 2 mA, Pd-thick filter; 50 kV, 2 mA, Cu-filter. Measuring time was 10 s for the 10 kV run and 20 s for the 30 kV and 50 kV runs. For the purpose of this study, we report the calcium and barium counts normalized to the sum of the eight most abundant elements in our records (Sum8), which are silicon, sulfur, potassium, calcium, titanium, iron, strontium and zirconium.

### Carbon, nitrogen and sulfur analyses

A total of 275 samples were collected with sampling resolution from 1 to 5 cm from the examined sediment cores. Prior to the measurement of the content and isotopic composition of organic carbon (TOC, δ^13^Corg) and bulk nitrogen (TN, δ^15^N), 0.3 g of dry sediments were weighed in 10 ml Eppendorf tubes and treated with 5 ml 6 N HCl to remove the carbonate component. Samples were rinsed five times with distilled water and let dry out in oven at 50 °C. Analysis were performed using a Thermo-Fisher MAT253 IRMS coupled to a Flash HT Plus Elemental Analyzer. Replicates of samples were run throughout the session of analysis and provided repeatability for TOC, TN, δ^13^C_org_ and δ^15^N better than 0.8%, 0.005%, 1‰ and 1‰, respectively. Certified reference material was measured every 25 samples and treated as unknown for quality control. δ^13^C and δ^15^N are determined by normalization to international scales VPDB (δ^13^C) and Air-N2 (δ^15^N) by three in-house standards. The C/N atomic ratio was calculated using the atomic mass weighted ratio of TOC and TN as C/N = (TOC/12.011)/(TN/14.007).

Total sulfur contents in sediment samples were measured on 0.23 g aliquots of unacidified material by combustion using a LECO CS744 (LECO, Michigan, USA). Blanks and drift standards 501–676 LNJ0370-1 and 501-505 LN I352 were run along with the samples for quality control. Measured standards agreed with the certified values for both carbon and sulfur.

### Radiocarbon dating

Radiocarbon analyses were conducted on four shells collected from core 1145GC (at 45 cm), 1143GC (at 82 cm and 160 cm) and 1174GC (at 45 cm) and one sample composed of mixed foraminifera collected from gravity cores 1145GC (at 101 cm). All specimens were collected away from the bottom and top of the cores in order to avoid reworked material and away from modern SMTZ to exclude potential contamination from methane-derived carbonate overgrowth. Foraminiferal tests were hand-picked from the coarse fraction (> 64 µm) of the sediments. Analyses were conducted at Beta Analytic Ltd (Miami, USA) and calibrated radiocarbon ages were obtained using the database MARINE13^72^. Conventional radiocarbon ages were adjusted for local reservoir correction (Delta-R = 67 ± 34).

### Pore water and headspace gas analyses

Pore water samples from the examined sediment cores were collected at a resolution of 1 cm to 15 cm. Pore water was collected using rhizons. Samples were transferred to 5 ml Eppendorf Tubes and kept frozen at −20 °C for onshore sulfate analysis. Sulfate concentration was determined via ion chromatography on a Metrosep A Supp 4 column at the University of Bergen, Department of Earth Science (Bergen, Norway). Repeated measurements of ERM CA016a certified material were used to check the accuracy and precision during the analyses: measured values agree within the uncertainty of certified value. Around 5 mL of sediment was collected for headspace gas analysis from the bottom of the cores or at different stratigraphic levels. To increase the number of gas samples we included data from additional cores collected from the Ploughmark area for a total of 27 samples (Supplementray Table S3). Sediment samples were collected using a syringe without the luer tip. Material was transferred to a 20 mL serum vial containing a glass bead and 5 mL of 1 M NaOH was added of to stop microbial activity. The vial was immediately closed with a septum and an aluminium crimp seal and stored at 4 °C. Analyses were conducted at the Applied Petroleum Technology (APT, Oslo) laboratory following the standard procedures from NIGOGA (Norwegian Industry Guide to Organic Geochemical Analysis)^73^. The carbon isotopic composition of the hydrocarbon gas components and the hydrogen isotopic composition of methane were determined using a Trace 1310 gas chromatograph (Thermo Fisher Scientific) equipped with a Poraplot Q column and PTV injector. The GC is interfaced via GC-Isolink II and Conflo IV to a Delta V Isotope Ratio Mass Spectrometer (IRMS) (Thermo Fisher Scientific). Repeatability based on analysis of standards was better than 1‰ VPDB for δ^13^C and better than 10‰ VSMOW for δD.

### Mineralogical and C, O isotopic analysis (MDAC and foraminifera)

Methane-derived authigenic carbonates were collected from core 1143GC at the sediment interval 4–6 cm and from 1141MC at 18–20 cm and 26–28 cm. Samples were rinsed with distilled water to remove salts and sediments, and then dried at 60°. Each sample was crushed and homogenized by hand grinding in agate mortar. Measurements were conducted at Centro Interdipartimentale Grandi Strumenti (C.I.G.S.) of the University of Modena and Reggio Emilia, Italy, using a PANalytical X’Pert PRO diffractometer (Malvern Panalytical Ltd., UK) with Cu Kα radiation (1.54 Å λ, operated at 40 kV and 40 mA) and equipped with an X’Celerator detector. Data were collected in the angular range 3º to 80º 2θ at 0.02 º/s and goniometry speed of 50 s/step. Semiquantitative phase analysis was performed using the software GSAS. The Mg-content (mol %) in calcite was calculated by applying the equation of Titschack et al.^74^ based on the value of calcite cell-volume (V). Calcite with mol% MgCO_3_ > 5 is considered high magnesium calcite (high-Mg calcite). Variations in the Mg content of the foraminiferal tests were semi-quantitatively assessed via SEM–EDS analysis of carbon-coated specimens using a SEM Hitachi Tabletop Microscope TM-3030 equipped with a Bruker Quantax 70 EDS Detector. These data were used to identify Mg-calcite overgrowth related to AOM.

Carbon and oxygen stable isotope analyses were conducted on MDAC and foraminiferal samples collected along the gravity cores 1143GC, 1145GC and 1174GC maintaining, where possible, a sampling resolution of 10 cm. For each sediment sample, around 15 tests of benthic foraminifera *Melonis barleeanus* were hand-picked from the coarse fraction (> 63 µm) of sediments, providing a total of 40 samples. MDAC and foraminifera samples were reacted with anhydrous phosphoric acid for 3 h at 50 °C and the resulting CO_2_ analysed on a Thermo Scientific Gasbench II coupled to a Finnigan MAT 253 triple collector isotope ratio mass spectrometer. Data are reported in‰ notation relative to VPDB. Analytical error was better than 0.1‰ (1SD) for both carbon and oxygen.

### Modelling of the Gas Hydrate Stability Zone

The paleo and modern gas hydrate stability zones in the Pockmark and Ploughmark areas were calculated assuming steady state using the graphical user interface CAGEHYD^75^ based on the CSMHYD code^64^. We calculated the GHSZ in the Pockmark area at ~ 15 cal ka BP assuming average water depths similar to present-day values^23^, salinity = 35 psu and bottom water temperature of 1 °C^76^. To model the modern GHSZ in the Ploughmark area we assumed a bottom water temperature of 2.5 °C^68^. In both models, we used the gas composition measured in exploration well 7321 8–1, 99–98% methane, 1–2% ethane (http://factpage.npd.no). The geothermal gradient used in this model is 38 °C/km^77^.

### Supplementary Information

Supplementary Information.

## Data Availability

All data generated or analysed during this study are included in this published article (and its Supplementary Information files).
