# Author Correction: Dynamic and history of methane seepage in the SW Barents Sea: new insights from Leirdjupet Fault Complex

**DOI:** 10.1038/s41598-024-57482-4

**Published:** 2024-03-25

**Authors:** Claudio Argentino, Kate Alyse Waghorn, Sunil Vadakkepuliyambatta, Stéphane Polteau, Stefan Bünz, Giuliana Panieri

**Affiliations:** 1 CAGE - Centre for Arctic Gas Hydrate, Environment and Climate, Department of Geosciences, UiT The Arctic University of Norway, 9037 Tromsø, Norway; 2 Oslo Innovation Center, VBPR - Volcanic Basin Petroleum Research, 0349 Oslo, Norway; 3 Institute for Energy Technology, 2007 Kjeller, Norway; 4 SurfExGeo, 0776 Oslo, Norway

Correction to: *Scientific Reports* 10.1038/s41598-021-83542-0, published online 23 February 2021

The original version of this Article contained errors in the Methods section, under the subheading ‘Pore water and headspace gas analyses’, where it was incorrectly stated that pressure filtration was conducted. As a result,

“Pore water was collected using rhizons and pressure filtration through 0.2 µm cellulose acetate filters.”

now reads:

“Pore water was collected using rhizons.”

Additionally, an incorrect analytical instrument was described for the determination of the sulfate concentration.

“Sulfate concentration was determined via inductively coupled plasma—optical emission spectrometry using a Thermo Scientific iCAP7000 hosted at the University of Bergen, Department of Earth Science (Bergen, Norway).”

now reads:

“Sulfate concentration was determined via ion chromatography on a Metrosep A Supp 4 column at the University of Bergen, Department of Earth Science (Bergen, Norway).”

Furthermore, Figure 4 contained errors where the samples in panels A and C were shifted from their correct positions in the plots.

The original Figure 4 and accompanying legend appear below.

**Figure 4:**
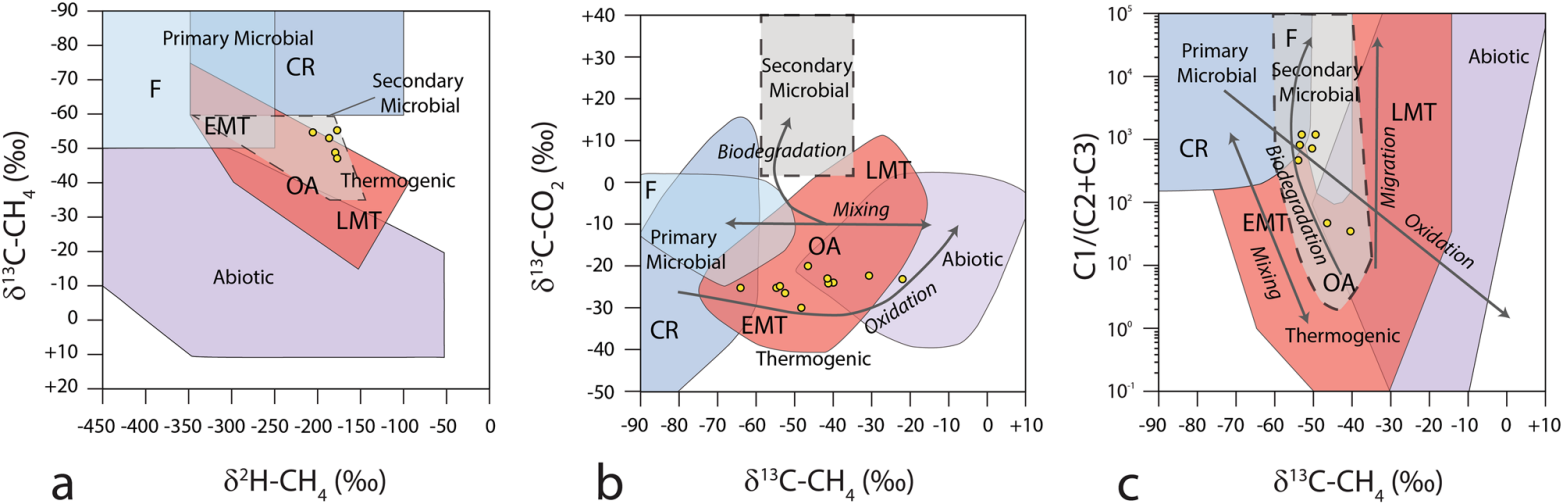
(**a**) Stable carbon (δ^13^C) and hydrogen (δ^2^H) isotope composition of methane from headspace gas analysis. Samples are reported in yellow dots. Genetic fields (CR -CO_2_ reduction, F—methyl-type fermentation, EMT—early mature thermogenic gas, OA—oil-associated thermogenic gas, LMT—late mature thermogenic gas) after Milkov and Etiope^59^. (**b**) Genetic diagrams of δ^13^C-C1 versus δ^13^C-CO_2_. Grey arrows indicate the main processes that affect the carbon isotopic composition of methane and CO_2_ in natural gases^59^. (**c**) Plot of δ^13^C-C1 versus the composition of light hydrocarbon components (C1/(C2 + C3) ratio). Grey arrows indicate the main processes affecting δ^13^C-C1 and the molecular composition of gases^59^.

The original Article has been corrected.

