# Supplementary material for: Dynamic and history of methane seepage in the SW Barents Sea: new insights from Leirdjupet Fault Complex
Source: Sci Rep. 2021 Feb 23;11:4373. doi: 10.1038/s41598-021-83542-0 (PMC7902819; doi:10.1038/s41598-021-83542-0)
Supplement: Supplementary file 1 — Supplementary Information. [file 41598_2021_83542_MOESM1_ESM.pdf]

Dynamic and history of methane seepage in the SW Barents Sea: new insights from Leirdjupet Fault Complex

Claudio Argentino[1], Kate Alyse Waghorn[1], Sunil Vadakkepuliyambatta[1], Stéphane Polteau[2, 3, 4], Stefan Bünz[1], Giuliana Panieri[1]

[1]CAGE - Centre for Arctic Gas Hydrate, Environment and Climate, Department of Geosciences, UiT The Arctic University of Norway, 9037 Tromsø, Norway.

[2]VBPR - Volcanic Basin Petroleum Research, Oslo Innovation Center, N-0349 Oslo, Norway.

[3]Institute for Energy Technology, 2007 Kjeller, Norway.

[4]SurfExGeo, 0776 Oslo, Norway.

* Corresponding Author: Claudio Argentino claudio.argentino@uit.no

Supplementary Fig.S1: Plots of organic matter parameters, pore water sulfate and headspace methane concentration for all the examined sediment cores.

Supplementary Fig.S2: Sedimentological composition of gravity cores 1143GC, 1145GC, 1174GC and magnetic susceptibility data.

Supplementary Table S3: Full sediment geochemistry dataset.

Supplementary Table S4: Mineralogical and isotopic composition of methane-derived authigenic carbonates.

Supplementary Table S5: Molecular and isotopic composition of headspace gas samples.

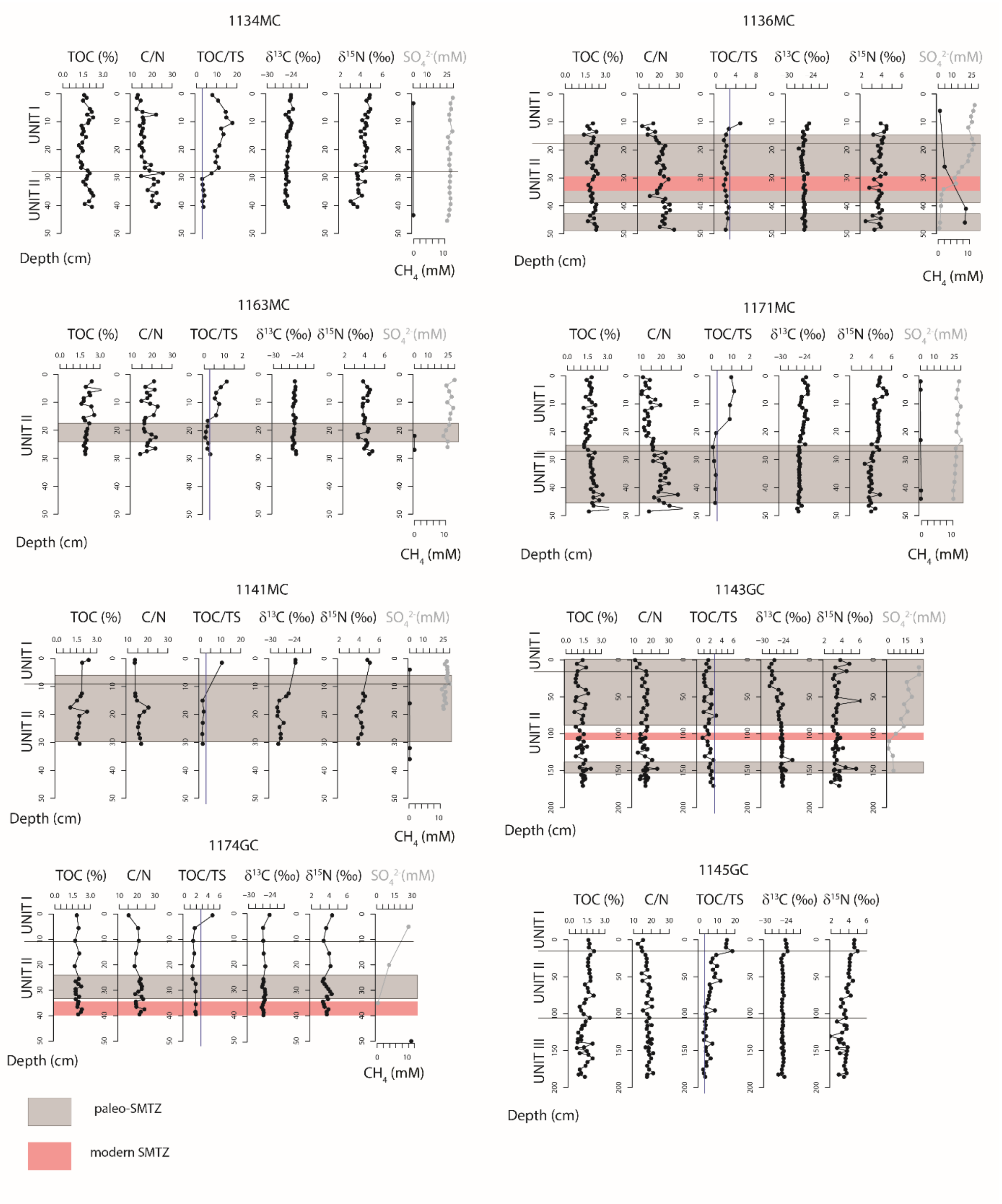

Supplementary Fig.S1. Geochemical composition of organic matter in the examined sediment cores and pore water sulfate and methane concentrations. The location of paleo and modern SMTZs are shown in grey and red color, respectively. Paleo-SMTZs in multicores 1136MC, 1163MC, 1171MC are identified based on sediment sulfur enrichments (TOC/TS < 2.8).

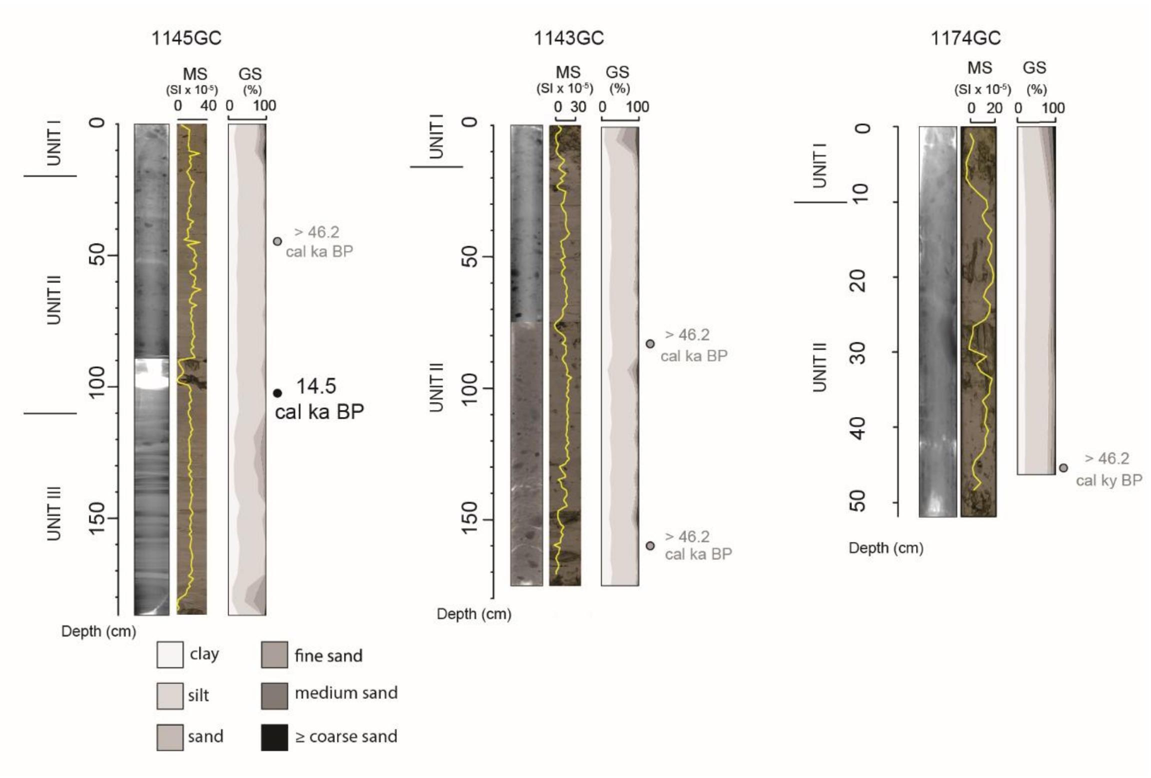

Supplementary Fig.S2. Lithology and magnetic susceptibility of gravity cores discussed in this study. For each core, X-Ray scan, Sidescan photographs and grain size data are reported. Description of lithological units UNIT I, UNIT II and UNIT III is provided in the manuscript. Radiocarbon dating results are also reported at the corresponding sampling position. MS : magnetic susceptibility; GS : grain size.

Core	Sample depth (cm)	$\delta^{13}C_{org}$ (‰)	TOC (wt. %)	$\delta^{15}N$ (‰)	TN (wt. %)	TC (wt. %)	TS (wt. %)	C/N	TOC/TS
1134MC	0-1	-24.1	1.6	4.9	0.1	2.3	0.2	12.9	7.8
	1-2	-24.1	1.8	4.9	0.2	-	-	12.5	-
	2-3	-24.4	1.5	4.6	0.1	2.1	0.1	14.3	10.6
	4-5	-	-	-	-	2.1	0.2	-	-
	5-6	-23.5	2.0	5.0	0.2	-	-	12.3	-
	6-7	-24.6	2.2	4.8	0.2	2.0	0.1	15.3	14.5
	7-8	-25.4	1.8	4.5	0.1	-	-	22.0	-
	8-9	-24.6	2.3	4.7	0.2	2.0	0.2	15.4	14.7
	9-10	-24.3	2.0	4.2	0.1	-	-	15.8	-
	10-11	-23.9	1.9	4.5	0.1	2.0	0.1	15.7	17.8
	11-12	-24.2	1.4	4.6	0.1	-	-	13.9	-
	12-13	-24.3	1.5	4.4	0.1	2.0	0.1	15.5	12.2
	13-14	-24.7	1.6	4.0	0.1	-	-	15.5	-
	14-15	-25.1	1.4	4.5	0.1	1.8	0.1	14.6	13.3
	15-16	-25.2	1.7	4.1	0.1	-	-	17.0	-
	16-17	-	-	-	-	1.9	0.1	-	-
	17-18	-24.4	1.5	4.4	0.1	-	-	14.9	
	18-19	-24.4	1.2	4.5	0.1	1.7	0.1	13.7	11.3
	19-20	-24.6	1.4	4.7	0.1	-	-	15.0	-
	20-21	-24.9	1.5	4.6	0.1	2.1	0.2	15.8	9.1
	22-23	-24.9	1.1	4.4	0.1	1.5	0.1	14.8	10.2
	24-25	-25.0	1.3	4.6	0.1	1.9	0.1	16.4	9.4
	25-26	-25.2	1.5	3.5	0.1	-	-	19.5	-
	26-27	-24.9	1.4	4.6	0.1	1.7	0.1	16.4	11.1
	27-28	-25.5	1.8	4.2	0.1	-	-	18.8	-
	28-29	-25.3	2.0	3.6	0.1	2.1	0.3	21.6	6.1
	29-30	-25.2	1.8	3.7	0.1	-	-	14.6	-

	30-31	-25.3	1.8	3.7	0.1	2.3	0.6	20.8	2.8
	31-32	-24.7	1.5	4.5	0.1	-	-	23.0	-
	32-33	-25.3	1.7	3.8	0.1	2.1	0.6	20.8	3.0
	34-35	-25.4	2.0	3.8	0.1	2.1	0.6	17.8	3.4
	35-36	-25.4	2.1	3.8	0.1	-	-	22.3	-
	36-37	-25.0	2.3	4.2	0.1	2.2	0.6	20.3	3.9
	38-39	-25.6	2.0	3.0	0.1	2.2	0.7	19.9	2.9
	39-40	-25.4	1.6	3.3	0.1	-	-	23.3	-
	40-41	-24.8	2.2	3.7	0.1	2.2	0.6	21.9	3.4
1136MC	10-11	-24.5	2.1	3.9	0.1	2.7	0.4	17.1	4.8
	11-12	-25.1	1.6	4.5	0.2	-	-	11.6	-
	12-13	-24.9	1.8	4.5	0.1	2.3	0.7	14.4	2.6
	13-14	-26.0	2.3	4.2	0.2	-	-	18.0	-
	14-15	-25.7	1.4	3.2	0.1	2.5	0.7	14.3	2.0
	15-16	-26.1	2.1	4.2	0.1	-	-	18.1	-
	16-17	-25.4	2.1	4.0	0.1	2.5	1.3	18.7	1.6
	17-18	-25.9	1.9	4.0	0.1	-	-	19.9	-
	18-19	-25.9	2.5	3.6	0.1	2.2	1.2	23.1	2.1
	19-20	-27.1	2.3	3.9	0.1	-	-	22.0	-
	20-21	-26.0	2.3	3.7	0.1	2.3	1.2	21.2	1.9
	21-22	-26.0	2.3	3.9	0.1	-	-	21.7	-
	22-23	-26.5	2.0	3.8	0.1	2.2	1.1	19.9	1.8
	23-24	-26.4	1.9	3.0	0.1	-	-	20.3	-
	24-25	-26.3	1.6	3.2	0.1	2.5	1.3	18.8	1.2
	25-26	-26.2	2.1	3.9	0.1	-	-	22.0	-
	26-27	-26.1	2.0	3.7	0.1	2.5	1.2	22.7	1.6
	27-28	-26.0	2.3	3.6	0.1	-	-	20.8	-
	28-29	-24.9	2.4	4.4	0.1	2.6	1.1	20.4	2.2
	29-30	-25.8	2.1	3.9	0.1	-	-	22.3	-

	32-33	-25.6	1.7	3.9	0.1	2.7	1.1	21.1	1.6
	33-34	-25.9	1.9	2.8	0.1	-	-	20.3	-
	34-35	-25.4	1.8	3.7	0.1	2.5	1.0	18.9	1.9
	35-36	-25.6	2.0	4.0	0.1	-	-	19.1	-
	36-37	-25.9	1.9	3.8	0.1	2.7	0.9	15.3	2.1
	37-38	-25.9	2.0	3.7	0.1	-	-	23.0	-
	38-39	-25.8	2.0	3.5	0.1	2.3	1.0	21.9	2.1
	39-40	-25.6	2.3	3.7	0.1	-	-	25.2	-
	40-41	-25.8	2.2	3.6	0.1	2.5	0.9	22.7	2.6
	41-42	-25.5	2.3	4.0	0.1	-	-	25.4	-
	42-43	-25.5	2.1	3.8	0.1	2.5	1.0	23.6	2.2
	43-44	-25.7	1.8	3.1	0.1	-	-	21.8	-
	44-45	-26.2	2.1	3.9	0.1	2.5	0.9	22.9	2.4
	45-46	-25.5	1.7	2.4	0.1	-	-	21.6	-
	46-47	-25.7	2.2	3.8	0.1	-	-	22.8	-
	47-48	-25.4	2.3	3.9	0.1	-	-	20.3	-
	48-49	-25.7	2.3	3.3	0.1	2.6	1.2	27.4	2.0
1141MC	0-1	-23.8	2.4	4.8	0.2	-	-	13.7	-
	1-2	-23.8	1.9	5.1	0.2	2.1	0.2	13.6	10.7
	12-13	-25.5	1.9	4.4	0.2	-	-	13.8	-
	13-14	-26.1	1.8	4.6	0.2	-	-	13.9	-
	14-16	-27.8	1.5	4.4	0.1	2.1	1.4	14.7	1.1
	17-18	-28.4	1.0	3.9	0.1	-	-	20.4	-
	18-20	-28.1	2.3	4.2	0.1	3.5	1.4	18.2	1.6
	20-21	-28.3	1.7	3.7	0.1	-	-	15.9	-
	22-24	-26.7	1.7	4.2	0.1	1.8	1.5	15.6	1.1
	24-25	-27.7	1.6	4.3	0.1	-	-	15.2	-
	26-28	-27.5	1.6	4.2	0.1	1.8	1.7	15.6	0.9
	28-29	-27.4	1.5	3.9	0.1	-	-	16.2	-

	30-31	-28.1	1.7	3.9	0.1	2.3	1.5	16.7	1.1
1143GC#1	0-1	-26.6	1.4	3.8	0.1	1.8	0.8	11.1	1.7
	5-6	-27.4	0.9	4.8	0.1	1.8	0.7	13.6	1.4
	10-11	-28.1	1.7	3.3	0.2	4.2	0.9	11.9	1.9
	15-16	-27.3	1.0	4.5	0.1	4.2	0.7	17.2	1.3
	20-21	-27.3	0.9	3.1	0.1	1.7	1.0	17.6	0.9
	25-26	-26.5	0.8	3.0	0.1	3.2	0.8	15.1	1.0
	30-31	-27.4	0.8	3.6	0.1	2.2	0.8	16.5	1.0
	35-36	-28.0	1.0	3.2	0.1	3.2	0.8	14.8	1.2
	40-41	-25.4	1.7	3.5	0.1	1.9	0.8	18.1	2.2
	45-46	-24.7	1.9	3.5	0.1	2.2	0.9	17.9	2.2
	50-51	-26.0	0.9	3.3	0.1	2.0	0.8	16.3	1.1
	55-56	-25.7	0.8	6.1	0.1	2.1	0.8	17.4	1.0
	60-61	-24.2	1.7	3.5	0.1	1.8	0.7	18.0	2.2
	65-66	-24.8	1.6	3.3	0.1	1.9	0.8	15.7	2.0
	70-71	-25.5	0.7	3.4	0.1	1.9	0.8	14.7	0.9
1143GC#2	0-1	-25.4	1.5	3.0	0.1	1.8	0.5	17.7	3.0
	5-6	-25.3	1.3	3.3	0.1	1.9	0.7	17.2	1.8
	10-11	-24.7	1.4	3.3	0.1	1.7	0.8	14.6	1.8
	15-16	-24.7	0.8	2.9	0.1	1.7	0.7	17.9	1.2
	20-21	-24.7	1.2	3.0	0.1	1.7	0.7	17.5	1.6
	25-26	-24.4	1.5	3.5	0.1	1.7	0.8	14.1	1.8
	30-31	-24.8	1.2	3.8	0.1	1.8	1.6	16.9	0.7
	31-32	-24.4	1.0	3.4	0.1	-	-	13.9	-
	32-33	-24.8	1.1	3.5	0.1	-	-	15.5	-
	35-36	-24.3	1.5	3.3	0.1	1.8	0.9	14.1	1.6
	40-41	-24.3	1.3	3.3	0.1	1.7	0.8	16.3	1.6
	42-43	-24.3	1.8	3.4	0.1	-	-	15.7	-
	43-44	-24.7	1.1	3.3	0.1	-	-	15.9	-

	44-45	-24.6	0.9	4.1	0.1	-	-	15.2	-
	45-46	-24.4	1.4	2.9	0.1	1.7	0.7	18.0	2.1
	46-47	-24.4	1.3	3.6	0.1	-	-	17.7	-
	50-51	-24.5	1.4	3.2	0.1	1.6	0.7	12.7	2.0
	55-56	-24.6	0.8	3.1	0.1	1.6	0.8	16.6	1.1
	60-61	-21.5	1.6	3.4	0.1	1.6	0.6	20.7	2.6
	65-66	-24.6	1.5	3.2	0.1	1.7	0.7	18.0	2.0
	70-71	-24.9	1.2	4.4	0.1	1.7	0.6	17.0	1.8
	72-73	-24.1	2.1	5.6	0.1	-	-	23.5	-
	73-74	-24.5	1.7	3.2	0.1	-	-	17.9	-
	74-75	-23.4	1.4	3.1	0.1	-	-	15.6	-
	75-76	-24.5	1.5	3.7	0.1	1.7	0.7	16.9	2.3
	80-81	-24.4	1.3	3.2	0.1	1.7	0.6	16.4	2.1
	81-82	-24.3	1.5	3.6	0.1	-	-	17.3	-
	82-83	-24.1	1.6	3.2	0.1	-	-	14.2	-
	83-84	-24.4	1.5	3.7	0.1	-	-	17.8	-
	84-85	-24.4	1.5	3.7	0.1	-	-	18.5	-
	85-86	-24.3	1.6	3.7	0.1	1.6	0.7	17.0	2.4
	86-87	-24.2	1.6	3.2	0.1	-	-	13.8	-
	87-88	-24.3	1.5	3.8	0.1	-	-	17.8	-
	90-91	-24.5	1.4	2.7	0.1	1.6	0.7	16.8	2.1
	95-96	-24.3	1.4	3.7	0.1	1.7	0.6	16.9	2.6
1145GC#1	0-1	-24.3	1.6	4.6	0.1	1.6	0.1	15.3	15.5
	5-6	-24.0	1.7	4.6	0.2	1.8	0.1	12.4	15.1
	10-11	-23.9	1.6	4.6	0.1	2.0	0.1	14.4	14.9
	15-16	-23.6	2.1	5.0	0.2	2.3	0.1	14.8	18.5
	20-21	-24.8	1.8	4.2	0.1	1.9	0.2	16.7	9.5
	25-26	-25.4	1.6	4.1	0.1	1.8	0.2	17.4	7.3
	30-31	-25.4	1.6	4.1	0.1	1.8	0.2	17.4	6.5

	35-36	-25.0	1.6	4.1	0.1	1.6	0.2	17.1	9.9
	40-41	-25.1	1.7	4.0	0.1	1.7	0.2	17.8	7.5
	45-46	-25.0	1.7	4.0	0.1	1.7	0.2	14.5	8.9
	50-51	-24.9	1.8	4.0	0.1	1.7	0.2	19.0	8.3
	55-56	-24.9	1.8	4.5	0.1	1.6	0.2	14.9	11.8
	60-61	-25.1	1.3	3.9	0.1	1.6	0.2	18.6	5.7
	65-66	-25.0	1.5	3.7	0.1	1.6	0.2	19.5	6.5
	70-71	-25.0	1.6	3.9	0.1	1.6	0.3	18.5	5.8
	75-76	-24.7	2.1	4.2	0.1	1.7	0.4	17.8	5.3
	80-81	-25.1	1.4	3.1	0.1	1.6	0.3	20.4	5.1
	85-86	-25.2	1.3	3.4	0.1	1.6	0.3	17.1	4.4
1145GC#2	0-1	-25.2	0.9	3.1	0.0	1.5	0.3	20.4	3.2
	5-6	-25.2	1.0	3.6	0.1	1.3	0.1	15.3	8.6
	10-11	-24.8	1.2	3.5	0.1	1.8	0.3	17.7	3.5
	15-16	-24.9	1.7	3.7	0.1	1.9	0.4	18.8	4.1
	20-21	-25.0	1.4	2.7	0.1	1.6	0.4	17.4	3.1
	25-26	-24.9	1.3	3.5	0.1	1.5	0.4	20.2	3.5
	26-27	-24.9	1.1	3.7	0.1	-	-	17.8	-
	30-31	-25.2	1.0	3.7	0.1	1.3	0.3	17.4	3.3
	35-35	-25.2	0.7	3.2	0.0	1.3	0.3	18.8	2.2
	40-41	-25.0	1.0	1.9	0.1	1.4	0.3	17.9	3.7
	44-45	-25.3	0.7	3.1	0.0	-	-	20.3	-
	45-46	-25.1	1.0	2.9	0.1	1.6	0.4	19.1	2.5
	48-49	-25.2	0.7	2.9	0.0	-	-	18.1	-
	50-51	-24.7	2.0	3.4	0.1	2.1	0.3	20.0	7.2
	55-56	-25.0	1.2	3.9	0.1	1.8	0.3	16.7	3.6
	56-57	-25.3	0.6	2.7	0.0	-	-	18.6	-
	60-61	-24.7	1.5	3.8	0.1	1.7	0.4	18.4	3.5
	63-64	-25.1	0.9	3.0	0.0	-	-	21.1	-

	65-66	-24.9	1.6	3.7	0.1	1.9	0.3	18.9	4.6
	70-71	-24.7	2.0	3.6	0.1	2.2	0.3	19.9	6.3
	75-76	-24.8	1.5	3.6	0.1	1.9	0.4	18.7	3.7
	80-81	-25.0	1.4	3.4	0.1	1.7	0.3	17.6	4.0
	85-86	-25.2	0.9	3.6	0.1	1.2	0.4	18.7	2.0
	90-91	-25.2	1.0	3.7	0.1	1.5	0.4	21.1	2.5
	92-93	-26.2	0.8	2.9	0.1	-	-	17.7	-
	95-96	-24.5	1.3	3.4	0.1	1.6	0.4	17.5	3.2
1163MC	0-1	-	-	-	-	2.1	0.2	-	-
	2-3	-24.7	2.4	3.9	0.1	2.1	0.2	21.0	11.2
	4-5	-24.5	1.9	4.3	0.1	2.3	0.2	16.7	8.3
	5-6	-24.6	3.3	4.6	0.2	-	-	21.3	-
	6-7	-24.9	2.2	4.4	0.1	2.2	0.4	17.5	5.9
	7-8	-24.7	2.3	4.3	0.1	-	-	18.9	-
	8-9	-25.0	1.8	3.9	0.1	2.4	0.3	17.6	5.3
	9-10	-24.5	1.8	4.3	0.1	-	-	14.7	-
	10-11	-24.9	1.6	3.8	0.1	2.2	0.2	19.1	7.5
	11-12	-25.2	2.4	3.8	0.1	-	-	22.8	-
	14-15	-25.1	2.6	3.9	0.1	2.1	0.4	20.9	6.0
	15-16	-24.5	1.8	4.1	0.1	-	-	16.8	-
	16-17	-24.9	1.9	4.0	0.1	2.1	1.0	16.2	1.8
	17-18	-24.7	2.2	4.0	0.2	-	-	16.2	-
	18-19	-	2.1*	-	-	2.5	1.2	-	1.7
	19-20	-24.9	2.1	4.4	0.2	-	-	15.9	-
	20-21	-24.9	2.0	4.3	0.1	2.1	2.2	16.4	0.9
	21-22	-24.9	2.0	3.3	0.1	-	-	19.7	-
	22-23	-25.4	1.9	3.4	0.1	1.9	3.0	21.9	0.6
	23-24	-25.1	2.0	4.1	0.1	-	-	19.1	-
	24-25	-24.8	1.9	4.3	0.1	2.1	0.9	17.9	2.0

	25-26	-25.2	1.8	4.2	0.1	-	-	17.6	-
	26-27	-25.2	2.0	4.0	0.1	2.2	1.2	21.7	1.6
	27-28	-24.4	1.9	4.8	0.1	-	-	17.4	-
	28-29	-24.4	1.9	4.5	0.2	2.2	0.6	14.1	3.1
1171MC	0-1	-23.6	1.8	4.8	0.2	2.1	0.2	11.0	9.8
	1-2	-24.4	1.4	4.8	0.1	-	-	14.4	-
	2-3	-23.9	1.7	4.8	0.2	-	-	12.2	-
	3-4	-23.2	1.7	4.6	0.2	-	-	12.5	-
	4-5	-23.4	1.8	5.2	0.2	-	-	14.1	-
	5-6	-23.2	1.8	5.5	0.2	2.1	0.2	10.6	11.3
	6-7	-23.2	1.7	5.5	0.2	-	-	10.3	-
	7-8	-24.7	1.4	5.0	0.1	-	-	13.4	-
	8-9	-24.8	1.6	4.3	0.1	-	-	17.2	-
	9-10	-24.9	1.8	4.4	0.1	-	-	14.6	-
	10-11	-25.2	2.1	4.2	0.1	2.2	0.2	19.6	9.2
	11-12	-24.5	1.2	4.3	0.1	-	-	15.4	-
	12-13	-23.6	1.8	4.8	0.2	-	-	12.1	-
	13-14	-23.9	1.8	4.7	0.2	-	-	13.3	-
	14-15	-24.0	1.6	4.6	0.1	-	-	13.5	-
	15-16	-23.3	2.1	4.7	0.2	2.3	0.2	11.7	9.0
	16-17	-24.1	1.7	4.5	0.1	-	-	14.1	-
	17-18	-24.2	1.4	4.7	0.1	-	-	13.9	-
	18-19	-24.4	1.4	4.7	0.1	-	-	11.5	-
	19-20	-24.5	1.5	4.7	0.1	-	-	11.9	-
	20-21	-24.4	1.4	4.8	0.1	1.7	0.6	13.7	2.3
	21-22	-25.1	1.6	4.8	0.2	-	-	12.8	-
	22-23	-24.9	1.5	4.7	0.1	-	-	16.2	-
	23-24	-25.2	1.3	4.6	0.1	-	-	15.9	-
	24-25	-23.9	1.2	4.0	0.1	-	-	15.4	-

	25-26	-25.4	1.3	4.5	0.1	1.3	1.9	16.1	0.7
	26-27	-25.3	1.5	4.8	0.1	-	-	15.9	-
	27-28	-25.3	2.0	4.1	0.1	-	-	22.3	-
	28-29	-25.6	1.6	4.0	0.1	-	-	16.8	-
	29-30	-25.2	1.9	4.0	0.1	-	-	20.4	-
	30-31	-25.4	1.7	4.2	0.1	2.0	1.4	16.0	1.2
	31-32	-25.1	1.7	3.3	0.1	-	-	21.5	-
	32-33	-25.2	1.7	3.9	0.1	-	-	22.0	-
	33-34	-25.4	1.9	3.9	0.1	-	-	23.7	-
	34-35	-25.4	1.8	3.7	0.1	-	-	22.4	-
	35-36	-25.3	2.0	4.0	0.1	2.0	0.9	19.7	2.2
	36-37	-25.3	1.8	3.8	0.1	-	-	22.2	-
	37-38	-25.2	1.9	3.9	0.1	-	-	19.6	-
	38-39	-25.4	2.0	4.0	0.1	-	-	23.8	-
	39-40	-25.3	2.2	3.7	0.1	-	-	19.5	-
	40-41	-25.0	1.8	4.0	0.1	2.3	0.9	19.9	1.9
	41-42	-25.4	1.9	3.8	0.1	-	-	18.7	-
	42-43	-25.6	2.6	4.8	0.1	-	-	28.4	-
	43-44	-25.3	1.9	3.7	0.1	-	-	16.5	-
	44-45	-25.5	2.4	-	-	-	-	-	-
	45-46	-25.5	1.9	4.0	0.1	2.2	1.0	21.5	1.9
	46-47	-24.7	2.0	3.7	0.1	-	-	24.2	-
	47-48	-25.8	4.1	4.4	0.1	-	-	32.4	-
	48-49	-25.5	1.6	4.0	0.1	-	-	14.0	-
1174GC	0-1	-24.1	1.9	4.3	0.1	2.1	0.4	15.2	4.8
	5-6	-26.0	2.0	3.7	0.1	2.2	1.2	20.6	1.7
	10-11	-25.9	1.7	3.4	0.1	2.0	1.2	21.0	1.5
	15-16	-25.8	2.1	4.0	0.1	2.2	1.2	19.4	1.7
	20-21	-25.3	1.7	4.2	0.1	2.4	1.2	18.6	1.4

25-26	-25.9	2.0	3.4	0.1	2.2	1.5	21.9	1.4
26-27	-25.8	1.8	3.3	0.1	-	-	21.2	-
27-28	-	2.0*	-	-	2.8	1.1	-	1.9
28-29	-26.2	2.2	3.7	0.1	-	-	22.7	-
29-30	-25.4	1.8	3.9	0.1	-	-	22.6	-
30-31	-	1.8*	-	-	1.7	0.9	-	1.9
31-32	-25.3	1.8	4.3	0.1	-	-	18.9	-
32-33	-25.7	2.1	3.7	0.1	-	-	22.5	-
33-34	-25.5	1.8	3.9	0.1	-	-	23.4	-
34-35	-25.8	2.0	3.3	0.1	-	-	19.2	-
35-36	-	1.9*	-	-	2.7	1.0	-	1.9
36-37	-26.3	1.9	3.5	0.1	-	-	19.6	-
37-38	-25.9	2.3	3.9	0.1	-	-	24.1	-
38-39	-25.8	2.2	3.7	0.1	3.8	1.2	22.1	1.8
39-40	-25.8	2.0	3.7	0.1	3.4	1.0	21.3	1.9

Supplementary Table S3. Geochemical composition of sediment samples collected from sediment cores examined in the present study. *average of nearest neighbours TOC values. Note that cores 1143GC and 1145GC are composed of 2 separate sections marked as #1 and #2.

Core	Sample depth (cm)	Cal (wt.%)	Mg (mol %)	Ara (wt.%)	Qtz (wt.%)	Feld (wt.%)	Chl (wt.%)	M/I (wt.%)	Gyp (wt.%)	$\delta^{13}C$ (‰)	$\delta^{18}O$ (‰)
1141MC	18-20	74.8	15.0	-	12.5	2.9	1.0	8.8	-	-31.0	5.3
	26-28	57.6	15.1	-	17.7	5.8	-	18.2	0.7	-31.5	5.0
1143GC	4-6	-	-	45.3	22.8	18.9	-	13.0	-	-30.0	5.0

Supplementary Table S4. Results from XRD and isotopic analyses on methane-derived authigenic carbonates. Cal: calcite; Ara: aragonite; Qtz: quartz; Feld: feldspars; Chl: chlorite; M/I: mica/illite; Gyp: gypsum.

Core	Geographic coordinates	C_1 (% THCG)	C_2 (% THCG)	C_{6+} (% THCG)	CO_2 (% THCG)	THCG (ppm)	$\delta^{13}C_{C1}$ (‰)	$\delta^{13}C_{C2}$ (‰)	$\delta^{13}C_{CO2}$ (‰)	δD_{C1} (‰)
1134MC	73° 54' 46.0" N<br>20° 55' 19.5" E	1.27	-	2.15	96.60	1005	nd	nd	-28.0	nd
1136MC	73° 54' 47.6" N<br>20° 55' 15.9" E	98.10	0.07	0.04	1.82	55397	-48.7	-23.2	-29.9	-150
1141MC	74° 00' 50.7" N<br>21° 04' 40.4" E	0.57	-	1.38	98.00	1935	nd	nd	nd	nd
1143GC	74° 00' 50.8" N<br>21° 04' 43.5" E	99.30	0.04	0.02	0.61	143368	-52.9	-25.8	-26.5	-158
1144GC	74° 00' 51.0" N<br>21° 04' 38.0" E	13.20	-	3.50	83.30	645	-64.4	nd	-25.1	nd
1145GC	74° 00' 19.2" N<br>21° 04' 13.0" E	1.37	-	3.41	95.20	575	nd	nd	nd	nd
1163GC	73° 54' 48.2" N<br>20° 55' 17.0" E	7.84	-	4.89	87.30	432	-40.4	nd	-24.0	nd
1164MC	73° 54' 48.5" N<br>20° 55' 9.5" E	96.90	-	0.04	3.070	62084	-46.9	-22.6	-19.9	-148
1170MC	73° 54' 48.4" N<br>20° 55' 13.6" E	28.10	-	5.38	66.50	527	-41.7	nd	-23.1	nd
1171MC	73° 54' 48.5" N<br>20° 55' 12.1" E	37.80	-	3.14	59.10	734	-22.1	nd	-23.2	nd
1172GC	73° 54' 49.1" N<br>20° 55' 13.6" E	33.60	0.89	3.39	62.10	696	-41.5	-22.6	-24.0	nd
1174GC	73° 54' 48.3" N<br>20° 55' 14.4" E	99.20	0.06	0.03	0.74	73255	-54.5	-23.5	-24.9	-179
1175GC	73° 54' 48.9" N<br>20° 55' 14.4" E	17.90	0.73	6.51	74.90	310	-30.8	nd	-22.2	nd
1176GC	73° 54' 48.3" N<br>20° 55' 18.2" E	97.90	0.12	0.17	1.77	14146	-55.2	-22.2	-25.1	-148

Supplementary Table S5. Geochemical characterization of hydrocarbon gas and carbon dioxide in headspace gas samples collected from sediment cores at Leirdjupet Fault Complex. nd: not determined.
